# Role of Microbiota-Modified Bile Acids in the Regulation of Intracellular Organelles and Neurodegenerative Diseases

**DOI:** 10.3390/genes14040825

**Published:** 2023-03-29

**Authors:** Yoshimitsu Kiriyama, Hiromi Nochi

**Affiliations:** 1 Kagawa School of Pharmaceutical Sciences, Tokushima Bunri University, Kagawa 769-2193, Japan; 2 Institute of Neuroscience, Tokushima Bunri University, Kagawa 769-2193, Japan

**Keywords:** bile acids, mitochondria, autophagy, Alzheimer’s disease, Parkinson’s disease, Huntington’s disease, neurodegeneration, neurodegenerative disease

## Abstract

Bile acids (BAs) are amphiphilic steroidal molecules generated from cholesterol in the liver and facilitate the digestion and absorption of fat-soluble substances in the gut. Some BAs in the intestine are modified by the gut microbiota. Because BAs are modified in a variety of ways by different types of bacteria present in the gut microbiota, changes in the gut microbiota can affect the metabolism of BAs in the host. Although most BAs absorbed from the gut are transferred to the liver, some are transferred to the systemic circulation. Furthermore, BAs have also been detected in the brain and are thought to migrate into the brain through the systemic circulation. Although BAs are known to affect a variety of physiological functions by acting as ligands for various nuclear and cell-surface receptors, BAs have also been found to act on mitochondria and autophagy in the cell. This review focuses on the BAs modified by the gut microbiota and their roles in intracellular organelles and neurodegenerative diseases.

## 1. Introduction

**Figure 1:**
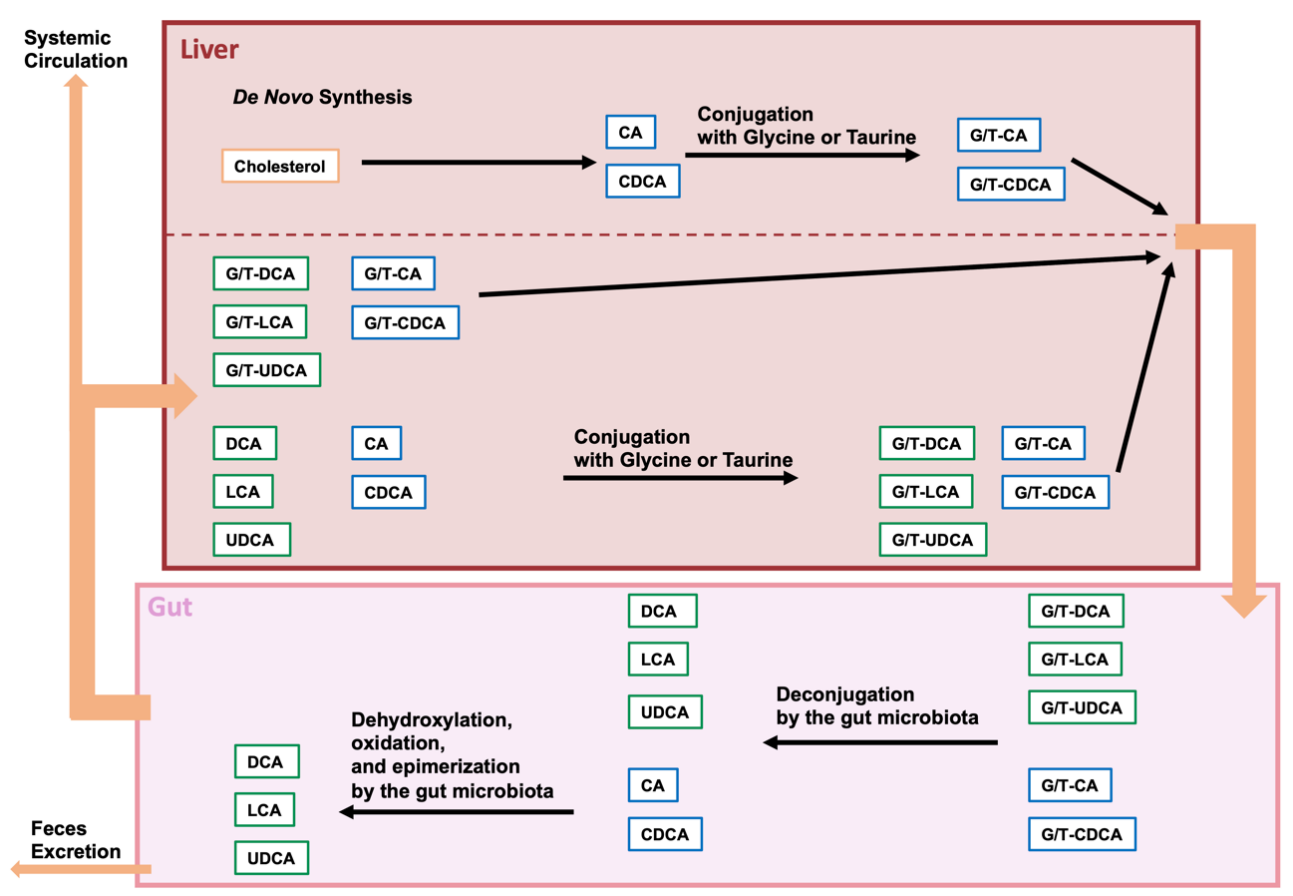
Modification of bile acids by the gut microbiome. Cholesterol is converted in the liver to cholic acid (CA) or chenodeoxycholic acid (CDCA), and these bile acids are then con-jugated with glycine or taurine (G/T). Conjugated CA and CDCA are transferred to the gallbladder and secreted from the gallbladder into the intestine upon food intake. Conjugated BA is deconjugated by intestinal bacterial bile salt hydrolases (BSHs), and CA and CDCA are further dehydrogenated to deoxycholic acid (DCA) and lithocholic acid (LCA), respectively. CDCA is also dehydrogenated and epimerized by intestinal bacteria and converted to ursodeoxycholic acid (UDCA). Approximately 95% of the BAs in the gut are absorbed and transferred to the liver, while the remaining BAs are excreted in the feces. Some of the BAs reabsorbed from the gut are effluxed into the systemic circulation.

Bile acids (BAs) are important amphiphilic steroidal molecules generated from cholesterol in the liver and are important components of bile. BAs that are produced by de novo synthesis in the liver are called primary BAs. BAs move from the liver to the gallbladder and are secreted from the gallbladder into the small intestine in response to food intake. Because BAs are amphiphilic, they can function as surfactants to form micelles with cholesterol, lipids, and lipophilic vitamins in the intestine, facilitating the digestion and absorption of these fat-soluble substances [1,2]. Most of the BAs that are involved in facilitating fat digestion and absorption are absorbed before passing through the ileum [3]. However, some BAs that are not taken up by the intestine are transferred to the colon. During their transit to the colon, BAs undergo various modifications by the gut microbiota and microbiota-modified bile acids are called secondary BAs [4]. Because BAs are modified in a variety of ways by different types of bacteria present in the gut microbiota, changes in the gut microbiota can affect the metabolism of BAs in the host [5,6]. Some of the modified BAs are then absorbed. The absorbed BAs in the gut are then transported to the liver via the portal vein. This circulation of BAs between the liver and the intestine is called enterohepatic circulation. However, the BAs that are not absorbed in the gut are ultimately egested in the feces. Therefore, BAs play a role in the excretion of cholesterol. Furthermore, although most BAs absorbed from the gut are transferred to the liver, some are transferred to the systemic circulation [6,7] (Figure 1).

BAs have also been found in the brain and are thought to migrate into the brain through the systemic circulation. Thus, there is a possible link between BA and neurological function and neurological disease [8,9]. Not only do BAs affect a variety of physiological functions by acting as ligands for various nuclear and cell-surface receptors [6], but BAs also affect mitochondria [10,11,12] and autophagy [13,14,15]. This review focuses on the BAs modified by the gut microbiota and their functions in intracellular organelles and neurodegenerative diseases.

## 2. BAs and Gut Microbiota

### 2.1. Bile Acid Modification by the Gut Microbiota

BAs are biosynthesized from cholesterol in the liver, and the biosynthesis of BAs is conducted via using the classical (or neutral) or alternative (or acidic) pathway, using more than 16 enzymes [16,17,18,19]. In the classical pathway, cholesterol is converted to 7α-hydroxycholesterol with cytochrome P450 (CYP) 7A1 (CYP7A1), and 7α-hydroxycholestero is then converted to 7α-hydroxy-4-cholesten-3-one. Cholic acid (CA) is biosynthesized from 7α-hydroxy-4-cholesten-3-one, involving CYP8B1, and chenodeoxycholic acid (CDCA) is biosynthesized from 7α-hydroxy-4-cholesten-3-one, involving CYP27A1. In the alternative pathway, cholesterol is metabolized to (25R)-26-hydroxycholesterol by CYP27A1, and this is then converted to CDCA by CYP7B1. Bile acid-CoA: amino acid N-acyltransferase (BAAT) conjugates CA and CDCA with glycine or taurine [20]. After the conjugation of glycine or taurine to BAs, these BAs are sent to and stored in the gallbladder. BAs are discharged into the small intestine through the stimulation of food intake [1]. 

In the intestine, bile salt hydrolase (BSH) deconjugates conjugated BAs. Various gut bacteria have been shown to exhibit BSH activity [21,22]. Most of these bacteria, such as *Lactobacillus*, *Bifidobacterium*, *Enterococcus*, *Clostridium*, and *Bacteroides* spp., are found in the ileum and colon [23,24,25,26]. After deconjugation, the 7α-hydroxy group is removed from CA and CDCA, leading to a yield of deoxycholic acid (DCA) and lithocholic acid (LCA), respectively. A few bacteria among *Clostridium* spp. have been identified as capable of eliminating the 7α-hydroxy group. The α-dehydroxylation of the CA and CDCA is carried out by enzymes that are encoded in the bile acid-inducible (*bai*) operon. In addition, CDCA is converted to ursodeoxycholic acid (UDCA) by 7α-hydroxysteroid dehydrogenase (7α-HSDH) and 7β-HSDH. In addition, DCA and LCA are converted to iso-DCA and iso-LCA by HSDHs, respectively [27].

### 2.2. Analysis of the Composition of the Gut Microbiota and Bacterial Species with Enzymes That Modify BAs

The composition of gut microbiota and BAs is affected by diet, age, sex, antibiotics, and disease [7,28,29,30]. BAs and gut microbiota affect each other [5,6]. BAs are modified by gut bacteria through a variety of enzymatic reactions. Thus, the diversity of gut bacteria involved in the modification of bile acids has implications for host physiology and pathophysiology. Bacteria harboring BSH have been reported to be widely distributed across bacterial phyla and approximately 26% of bacteria strains in the Human Microbiome Project [31]. However, bacteria capable of conducting 7-α-dehydroxylation is limited to *Clostridium* spp. [27,32,33]. Furthermore, changes in the levels of microorganisms in gut microbiota are associated with neurodegenerative diseases, including Alzheimer’s disease and Parkinson’s disease [34,35]. The relationship between gut microbiota and various diseases is currently being studied using multi-omics analysis, including metagenomics, metatranscriptomics, metaproteomics, and metabolomics [36]. There are two major types of metagenomic analyses that comprehensively analyze microbial communities using next-generation sequencers: 16S rRNA gene sequencing and shotgun metagenomic sequencing; these identify microorganisms and evaluate diversity and abundance [37]. 16S rRNA gene sequencing is the most widely used method of analyzing gut microbiota, and it is relatively inexpensive and simple to perform. In the 16S rRNA gene sequencing metagenomic analysis, after PCR amplification is conducted to target the 16S rRNA genes, the amplified PCR products are comprehensively sequenced using next-generation sequencing (NGS) to identify the diversity of gut microbiota and the types and composition ratios of its constituent bacteria. In shotgun metagenomic sequencing, DNA is randomly split into fragments, which are then sequenced by NGS. The sequenced DNA is linked using bioinformatics, resulting in the identification of species, strains, and functional genes [38,39]. These metagenomic analyses have revealed the association between changes in the composition of the gut microbiota and neurodegenerative diseases; furthermore, but their means, the association between changes in bacterial species with enzymes that modify BAs and neurodegenerative diseases are also being elucidated [34,35]. In patients with Alzheimer’s disease, Bacteroidetes is positively correlated with Alzheimer’s disease, while Firmicutes and *Bifidobacterium* are negatively correlated with Alzheimer’s disease [40,41,42]. In patients with Parkinson’s disease, *Akkermansia* is positively correlated with Parkinson’s disease, while *Lactobacillus* is negatively correlated with Parkinson’s disease [43,44,45]. In patients with Huntington’s disease, *Intestinimonas*, *Bilophila*, *Lactobacillus*, *Oscillibacter*, *Gemmiger*, and *Dialister* are positively correlated with Huntington’s disease [46] and Firmicutes, *Lachnospiraceae*, and *Akkermansiaceae* are negatively correlated with Huntington’s disease gene expansion carriers [47]. Furthermore, a recent study using shotgun metagenomic sequencing of Alzheimer’s disease patients has shown that *Bacteroides* spp., *Alistipes* spp., *Odoribacter* spp., and *Barnesiella* spp. increased and *Lachnoclostridium* spp. decreased in patients with Alzheimer’s disease [42]. Moreover, a recent study using shotgun metagenomic sequencing of Parkinson’s disease patients has shown that eighty-four species were associated with Parkinson’s disease, with 55 species more and 29 species less exist in Parkinson’s disease compared to healthy subjects. In addition, 34 genera more and 1 genus less present in Parkinson’s disease compared to healthy controls [48].

## 3. BAs as Regulatory Modulators

### 3.1. BAs as Regulatory Molecules of Nuclear and Cell-Surface Receptors

BAs are signaling molecules that bind to nuclear and cell-surface receptors, such as the farnesoid X receptor (FXR), a receptor for primary bile acids [49,50,51] and Takeda G protein receptor 5 (TGR5), also known as G protein-coupled bile acid receptor 1 and a receptor for secondary bile acids [50,51,52]. Among the nuclear receptors, BAs act as ligands for FXR, the constitutive androstane receptor, pregnane X receptor, vitamin D receptor, liver X receptor, glucocorticoid receptor, and retinoid-related orphan receptor γT. Moreover, BAs act as ligands for the cell-surface receptors TGR5, sphingosine-1-phosphate receptor 2, and the M2 and M3 muscarinic receptors. These receptors are expressed in various organs, including the brain [53,54,55,56,57,58]. Therefore, BAs play diverse physiological roles through the affect they have on these various receptors [7,59,60].

### 3.2. BAs as Regulatory Molecules of Intracellular Organelle

Mitochondrial and/or autophagic dysfunction affects neurodegenerative diseases, including Alzheimer’s disease, Parkinson’s disease, and Huntington’s disease [61,62,63].

**Figure 2:**
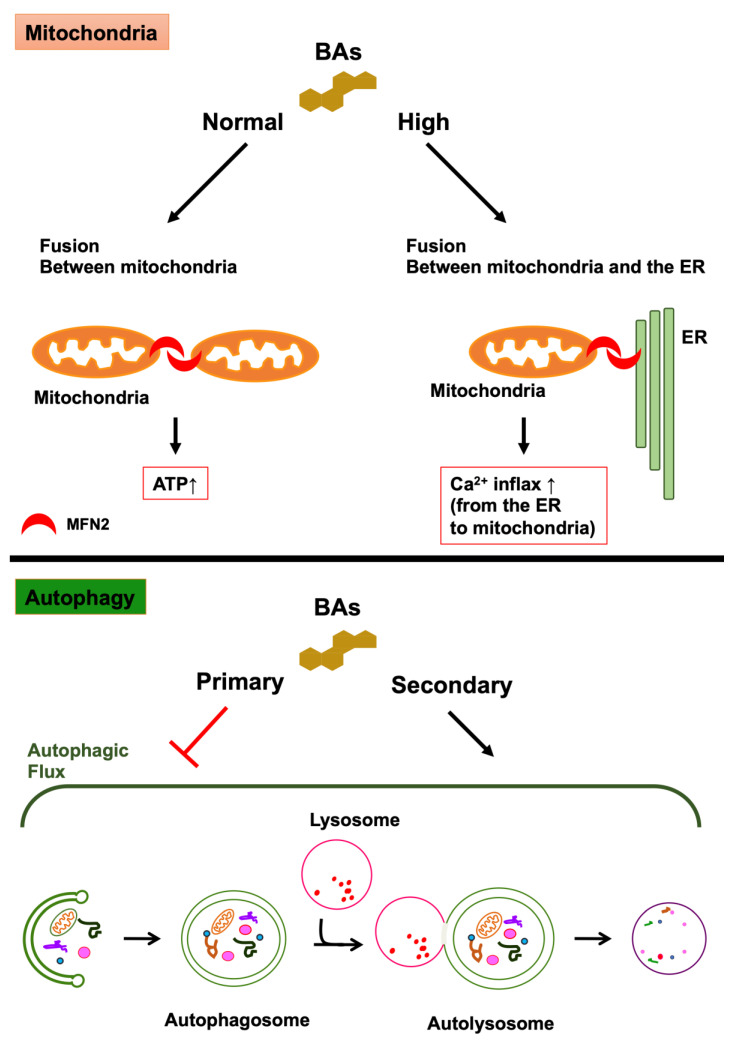
The effects of bile acids (BAs) on mitochondria and autophagy. The effects of BAs on mitochondria: DCA, CDCA, and their taurine conjugates activate MFN2 by binding directly to MFN2, promoting mitochondria-to-mitochondria fusion and mitochondria-to-ER fusion. These BAs, at normal concentration, promote the fusion between mitochondria, leading to the generation of ATP. By contrast, these BAs, at high concentration, promote the fusion between the mitochondria and the ER, leading to Ca2^+^ influx from the ER to the mitochondria. The effects of BAs on autophagy: secondary BAs (DCA and UDCA) induce autophagy and primary bile acids (CA, CDCA, and TCA) inhibit autophagy by preventing the formation of autolysosomes. In addition, the activation of TGR5, a receptor for secondary bile acids, leads to the activation of autophagy. By contrast, the activation of FXR, a receptor for primary bile acids, leads to the inhibition of autophagy.

Mitochondria are intracellular organelles that have a double membrane consisting of an inner and outer membranes and function in the process of cellular ATP synthesis. They are involved in diverse and varied cellular functions, including apoptosis, regulation of intracellular calcium concentration, and metabolism of glucose, fatty acids, and amino acids [64]. Mitochondria maintain a functional population by repeating fusion and fission to eliminate dysfunctional mitochondria, and mitochondrial dysfunction is involved in various types of diseases [65,66]. The equilibrium between fission and fusion plays a crucial role in mitochondrial quality control. The fusion of mitochondria causes an exchange of mitochondrial DNA (mtDNA) and metabolites between damaged and healthy mitochondria to avoid the accumulation of damaged substances into one mitochondrion. Mitochondrial fusion undergoes when the outer mitochondrial membranes (OMM) of two mitochondria fuse with each other and with the inner mitochondrial membranes. Mitofusin (MFN) plays a crucial role in the fusion of the outer membrane. In addition, optic atrophy 1 (OPA1) plays a crucial role in the fusion of the inner membrane [67,68,69]. MFN is a dynamin-like GTPase and consists of two isoforms, namely, MFN1 and MFN2. Although both isoforms have important roles to play in the tethering and fusion of OMMs, the tethering activities of MFN1 are higher than those of MFN2. Therefore, MFN1 is the main tethering isoform for the fusion of OMMs [70,71]. MFN2 is located on the mitochondria-associated endoplasmic reticulum (ER) membrane (MAM), and it connects mitochondria to the ER, bringing Ca^2+^ influx from the ER to the mitochondria [72,73]. Mitochondrial fission tends to isolate damaged DNA and metabolites in mitochondria. Dynamin-related protein 1 (DRP1) is the major regulator of mitochondrial fission. DRP1 forms a ring-like structure composed of multimer at mitochondrial fission sites of the OMM and causes the constriction and scission of mitochondria. The activity of DRP1 is controlled by post-translational modifications, such as phosphorylation, ubiquitination, sumoylation, S-nitrosylation, and O-GlcNAcylation [74]. Glycocholic acid (GCA), taurocholic acid (TCA), glycochenodeoxycholic acid (GCDCA), and taurochenodeoxycholic acid (TCDCA) are abundant in the mitochondria of the liver of healthy subjects [75]. However, most BAs are toxic to mitochondria at high concentrations and can lead to a decrease in mitochondrial membrane potential [11,76,77]. By contrast, tauroursodeoxycholic acid (TUDCA) enhances mitochondrial biogenesis and protects against mitochondrial dysfunction [11,12]. Moreover, peroxisome proliferator-activated receptor γ coactivator 1α, nuclear respiratory factor 1, and mitochondrial transcription factor A are related to mitochondrial biogenesis [65,78]. TGR5 activation induces these molecules and promotes the functional gain and biogenesis of mitochondria [79]. Therefore, drugs that activate TGR5, including BA, may be potential therapeutic agents for neurodegenerative diseases. It has been recently reported that DCA, CDCA, and their taurine conjugates activate MFN2 by binding directly to MFN2, promoting mitochondria-to-mitochondria fusion and mitochondria-to-ER fusion in THP-1 cells that are differentiating into macrophages [10] (Figure 2). DCA and CDCA at physiological conditions (5 μM) promote the fusion between mitochondria, resulting in an increase in the generation of ATP. By contrast, DCA and CDCA with cholestatic conditions (100 μM) enhance the fusion between the mitochondria and the ER, leading to Ca^2+^ influx from the ER to the mitochondria, the activation of NLRP3 inflammasome and pyroptosis, and innate immunity. Furthermore, CA and UDCA antagonize the effects of DCA and CDCA on the GTPase activity of MFN2. The areas where mitochondria and ER contact are called mitochondria-associated membranes or mitochondria-associated ER membranes (MAMs) and an association between MAMs and neurodegenerative diseases has been indicated [80,81]. MAMs are initiation sites of autophagosome formation [82,83]. In addition, knockdown of MFN2 evokes impaired autophagy [84,85]. By contrast, overexpression of MFN2 leads to the induction of autophagy [85]. In addition, it is critical to maintain a balance between mitochondrial fusion and fission to support both mitochondrial and cellular function, and mitochondrial structural changes are associated with neurodegenerative diseases. Because MFN2 plays a crucial role in controlling mitochondrial structure, MFN2 is related to neurodegenerative diseases [86].

Autophagosomes have an important role in autophagy. Autophagy is a major intracellular degradation process in which autophagosomes sequester cytoplasmic components such as damaged proteins and intracellular organelles and then fuse with lysosomes to degrade those cytoplasmic components. The intracellular organelles in which autophagosomes and lysosomes are fused together are called autolysosomes [87]. The mechanistic (or mammalian) target of rapamycin complex 1 (mTORC1) is the key regulator of the initiation phase of autophagy, although numerous other molecules, such as microtubule-associated protein 1 light chain 3 (LC3), are also involved in autophagy. The inhibition of mTORC1 leads to the activation of the Unc-51-like kinase 1/2 (ULK1/2), resulting in the conjugation of phosphatidylethanolamine to LC3 and the formation of autophagosomes [61,88]. The formation of an autolysosome in autophagosome-lysosome fusion is conducted by soluble N-ethylmaleimide-sensitive factor attachment protein receptors (SNAREs) [89,90], Rab7 [91,92], UV radiation resistance-associated gene protein (UVRAG) [93], the homotypic fusion and protein sorting (HOPS) complex [94], and LC3s [95,96]. DCA induces autophagy in the human esophageal epithelial cell line [14], and UDCA also induces autophagy in the human liver [15]. By contrast, GCDCA inhibits autophagy by suppressing transcription factor E3 (TFE3) in the human liver cell line [97]. Moreover, CA, CDCA, and TCA interfere with the formation of autolysosomes in hepatocytes and the liver [13,15]. The activation of FXR, a receptor for primary bile acids, leads to the inhibition of autophagy [98,99]. By contrast, the activation of TGR5, a receptor for secondary BAs, leads to the activation of autophagy [100] (Figure 2). The activation of TGR5 leads to the activation of cAMP-response element binding protein (CREB). CREB promotes autophagy and upregulates autophagy-related genes such as *Atg7*, *Ulk1*, and *Tfeb*, while FXR represses autophagy and the expression of these genes by binding to CREB [98]. Furthermore, CREB-regulated transcription coactivator 1 induces autophagy-related genes by binding to CREB in neurons in competition with FXR [101].

## 4. Microbiota-Modified BAs in Neurodegenerative Diseases

The source of the BAs found in the brain has not been identified, but conjugated and unconjugated BAs have been found there [102,103,104]. BAs present in the brain may be produced there or migrate through the circulatory system. Primary BAs (CA and CDCA) are also produced by de novo synthesis in the brain. These primary BAs are responsible for the majority of cholesterol metabolism in the brain. This third pathway in the brain to produce primary BAs by de novo synthesis is called neural pathway [105]. Because secondary BAs are generated by the gut microbiota, the secondary BAs detected in the brain are thought to be transferred from the circulation. Furthermore, because a correlation has been identified between brain bile acid concentrations and serum bile acid concentrations, it is now believed that the majority of BAs in the brain migrate through circulation [106,107]. However, because there is neural pathway to BA synthesis, primary BAs produced by de novo synthesis in the brain may also play a role in the physiological and pathophysiological condition [105]. BAs are transported from the circulation, either through the blood–brain barrier (BBB) or through BA transporters [16,108]. Lipophilic BAs can pass through the BBB through passive diffusion. By contrast, hydrophilic BAs can pass through the BBB through transporters [109,110]. Therefore, the brain can be influenced by the gut microbiota via BAs.

### 4.1. Alzheimer’s Disease

Alzheimer’s disease is a progressive and irreversible neurodegenerative disease characterized by dementia, memory loss, and morphological changes in multiple regions of the brain. The pathological features of patients with Alzheimer’s disease are the accumulation of amyloid β peptide and tau protein entanglement in the brain [111]. Amyloid β peptide is produced from amyloid precursor protein (APP) with β- and γ-secretase [112,113]. The γ-secretase complex contains presenilin 1 (PS1). Autophagy refers to the process of removing the accumulation of misfolded proteins, and the suppression of autophagy is connected with Alzheimer’s disease [114]. Therefore, BAs can affect Alzheimer’s disease by influencing autophagy. LCA levels in plasma are higher in patients with Alzheimer’s disease. In addition, LCA levels in plasma increase by approximately 3 fold within 8–9 years from when healthy subjects develop Alzheimer’s disease [115]. By contrast, the levels of CA in plasma and the TCA levels in the brain are significantly lower in patients with Alzheimer’s disease [104]. However, the neuroprotective effect of TUDCA, which is a taurine-conjugated secondary BA, has been demonstrated in neurodegenerative disease [116]. TUDCA lowers amyloid β peptides and relieves memory deterioration in APP/PS1 double-knockout mice used as a model for Alzheimer’s disease [117,118]. The ratios for both unconjugated and conjugated secondary BAs, including LCA, GCDCA, taurodeoxycholic acid (TDCA), glycodeoxycholic acid (GDCA), and UDCA, to CA were higher in the brain of patients with Alzheimer’s disease [102]. Furthermore, the ratios of DCA, GDCA, TDCA, and glycolithocholic acid. GLCA) were significantly higher in the serum of patients with Alzheimer’s disease [119]. Therefore, increased secondary BAs may be associated with Alzheimer’s disease, and the status of the gut microbiome may affect the progression and suppression of Alzheimer’s disease. In addition, the increase in *Lactobacillus*, which has BSH that deconjugates conjugated-BAs, in feces is related to Alzheimer’s disease [120,121].

### 4.2. Parkinson’s Disease

Parkinson’s disease is a progressive neurodegenerative disease that manifests itself in resting tremor, muscle rigidity, bradykinesia, akinesia, and postural reflex disorder [122]. It results in the loss of dopaminergic neurons in the substantia nigra [123]. The dysfunction of phosphatase and tensin homolog (PTEN)-induced putative kinase 1 (PINK1) and Parkin is considered a major cause of parkisonisms [124], and this dysfunction results in the impairment of mitophagy, suggesting that the quality control of mitochondria plays an crucial role in the suppression of Parkinson’s disease [61,125]. BAs are associated with autophagy and the quality control of mitochondria. Furthermore, plasma levels of CA, DCA, TDCA, and GDCA in patients with Parkinson’s disease are significantly higher when compared with healthy subjects [126,127]. However, the plasma levels of GUDCA in patients with Parkinson’s disease are decreased [128]. Moreover, levels of DCA and LCA are higher in the appendix and LCA is higher in the ileum of patients with Parkinson’s disease [129]. 1-Methyl-4-phenyl-1,2,3,6-tetrahydropyridine (MPTP) and rotenone treatment to rodents are commonly used to model Parkinson’s disease. These chemicals prevent the function of complex I in mitochondria, causing oxidative stress and mitochondrial damage in neurons, leading to neurotoxicity and parkinsonism [130,131]. TUDCA protects dopaminergic neurons from MPTP-induced neurotoxicity [132]. It also suppresses MPTP-induced loss of dopaminergic neurons and mitochondrial membrane potential to improve the motor symptoms induced by MPTP [133]. Furthermore, rat models of Parkinson’s disease induced by rotenone show mitochondrial swelling, and the loss of mitochondrial cristae and UDCA improves these mitochondrial abnormalities [134]. In addition, several reports have indicated that BSH-containing bacteria, such as *Lactobacillus*, *Bifidobacterium*, *Enterococcus*, and *Bacteroides* spp., are increased in patients with Parkinson’s disease [34,35]. This indicates a relationship between unconjugated BAs and Parkinson’s disease.

### 4.3. Huntington’s Disease

Huntington’s disease is an autosomal-dominant neurodegenerative disease that manifests in cognitive disability, and psychiatric disturbance, and motor dysfunctions [135]. Huntington’s disease is caused by cytosine-adenine-guanine (CAG) expansion which encodes a polyglutamine at the N-terminus of huntingtin (HTT) [136]. HTT has a similar structure to the three autophagy proteins of yeast, Atg11, Atg23, and Vac8 [137,138] and acts as an autophagy initiator and enhancer [139]. The mutation of HTT leads to a reduction in mitophagy [140]. BAs are associated with mitophagy. In addition, 3-nitropropionic acid (3-NP) selectively damages neurons in the striatum and is involved in the development of Huntington’s disease. 3-NP inhibits succinate dehydrogenase in mitochondria and leads to the degeneration of the caudate-putamen [141,142]. TUDCA improves 3-NP-induced neural mitochondrial damage, neural cell death, and sensorimotor deficits [143]. Moreover, TUDCA ameliorates striatal apoptosis, cerebral and striatal atrophy, and sensorimotor deficits in R6/2 transgenic mice that carry the human huntingtin gene which has 144 CAG repeats and are commonly used as a model for Huntington’s disease [144].

## 5. Concluding Remarks

Primary BAs are synthesized in the liver and these BAs are modified by the involvement of various microorganisms present in the gut microbiota. Therefore, a variety of bile acids exist in the human body. Bile acids have been shown to exert diverse physiological effects by binding to various nuclear and cell-surface receptors. Therefore, bile acids have come to be recognized as signaling molecules. In addition, it has recently been reported that BAs are present in mitochondria and affect mitochondrial ATP synthesis. Furthermore, some BAs have been shown to act on mitochondrial fusion as ligands for MFN2, which is involved in the fusion between mitochondria and between mitochondria and ER. BAs have also been shown to regulate the progression of autophagy. Although the causes of neurodegenerative diseases are still incompletely elucidated, mitochondrial and mitophagy dysfunction in neurons is considered one of the causes of neurodegenerative diseases, including Alzheimer’s disease, Parkinson’s disease, and Huntington’s disease. Therefore, further elucidation of the function of BAs and the gut microbiota which modifies BAs may lead to new treatments for neurodegenerative diseases.
